# Hyperthermia Induces the ER Stress Pathway

**DOI:** 10.1371/journal.pone.0023740

**Published:** 2011-08-18

**Authors:** Xu Xu, Sounak Gupta, Wenli Hu, Barbara C. McGrath, Douglas R. Cavener

**Affiliations:** Department of Biology, Center for Cellular Dynamics, Pennsylvania State University, University Park, Pennsylvania, United States of America; Cincinnati Children's Hospital Medical Center, United States of America

## Abstract

**Background:**

The ER chaperone GRP78/BiP is a homolog of the Hsp70 family of heat shock proteins, yet GRP78/BiP is not induced by heat shock but instead by ER stress. However, previous studies had not considered more physiologically relevant temperature elevation associated with febrile hyperthermia. In this report we examine the response of GRP78/BiP and other components of the ER stress pathway in cells exposed to 40°C.

**Methodology:**

AD293 cells were exposed to 43°C heat shock to confirm inhibition of the ER stress response genes. Five mammalian cell types, including AD293 cells, were then exposed to 40°C hyperthermia for various time periods and induction of the ER stress pathway was assessed.

**Principal Findings:**

The inhibition of the ER stress pathway by heat shock (43°C) was confirmed. In contrast cells subjected to more mild temperature elevation (40°C) showed either a partial or full ER stress pathway induction as determined by downstream targets of the three arms of the ER stress pathway as well as a heat shock response. Cells deficient for *Perk* or *Gcn2* exhibit great sensitivity to ER stress induction by hyperthermia.

**Conclusions:**

The ER stress pathway is induced partially or fully as a consequence of hyperthermia in parallel with induction of *Hsp70*. These findings suggest that the ER and cytoplasm of cells contain parallel pathways to coordinately regulate adaptation to febrile hyperthermia associated with disease or infection.

## Introduction

The induction of GRP78/BiP by pharmacological reagents that perturb homeostasis in the endoplasmic reticulum led to the discovery of the ER stress response. Although, GRP78/BiP is a homolog of the hsp70 heat shock proteins, it is not induced by severe heat shock [1], [2], [3]. The functions of the rough ER, including protein folding, protein quality control, and trafficking of client proteins to the Golgi, are sensitive to changes in calcium and the redox state, which in turn are influenced by physiological changes in the rest of the cell and extracellular environment [4], [5]. Severe perturbations in calcium levels, redox state, or the amount or nature of client proteins can activate a cascade of events for the purpose of restoring ER homeostasis [6], [7]. Three ER resident proteins, PERK, ATF6, and IRE1, act as sensors of ER perturbations and act to mobilize the ER stress response. ATF6 and IRE1 are largely responsible for inducing the transcription of ER chaperone and folding genes (e.g. *Bip*, *Dnajc3*, and *Erp72*), while PERK has a dual role in temporarily repressing global protein synthesis and inducing the translation of proteins that help to coordinate the ER stress response [4], [5]. PERK and IRE1 also have important functions in regulating normal physiology and development that are unrelated to the ER stress response and involve other regulatory pathways [8], [9], [10], [11]. In contrast the heat shock response is initiated by the activation of the heat shock transcription factor (HSF) that stimulates the rapid induction of the classic heat shock genes [12], [13], [14], [15]. The heat shock genes encode chaperone proteins that help to protect cytoplasmic proteins from denaturation and assist with refolding of proteins. Hence their functions are similar to the ER chaperones that are increased in expression in lumen of the ER during ER stress. Global protein synthesis is inhibited by both heat shock and ER stress, and the mechanism of inhibition is largely mediated by phosphorylation of eIF2α which results in the inhibition of recycling of eIF2-GDP necessary for new rounds of translation initiation [16], [17], [18], [19], [20], [21]. In the case of ER stress the recovery of protein synthesis is regulated by *Gadd34* [19], [20], [21], a regulatory subunit of protein phosphatase-1, which targets the dephosphorylation of eIF2α. *Gadd34* is likely to play an important role in regulating the recovery from heat shock as well but this has not been demonstrated.

We speculated that the ER does experience stress during heat shock, but this stress had previously gone undetected because the very high temperatures typically used in heat shock experiments resulted a global repression of transcription and translation [17], [18], [22], [23]. In particular, we surmised that the response to a milder heat shock that corresponds to the normal febrile-range hyperthermia over several hours might reveal an ER stress response.

## Results

**Figure 1:**
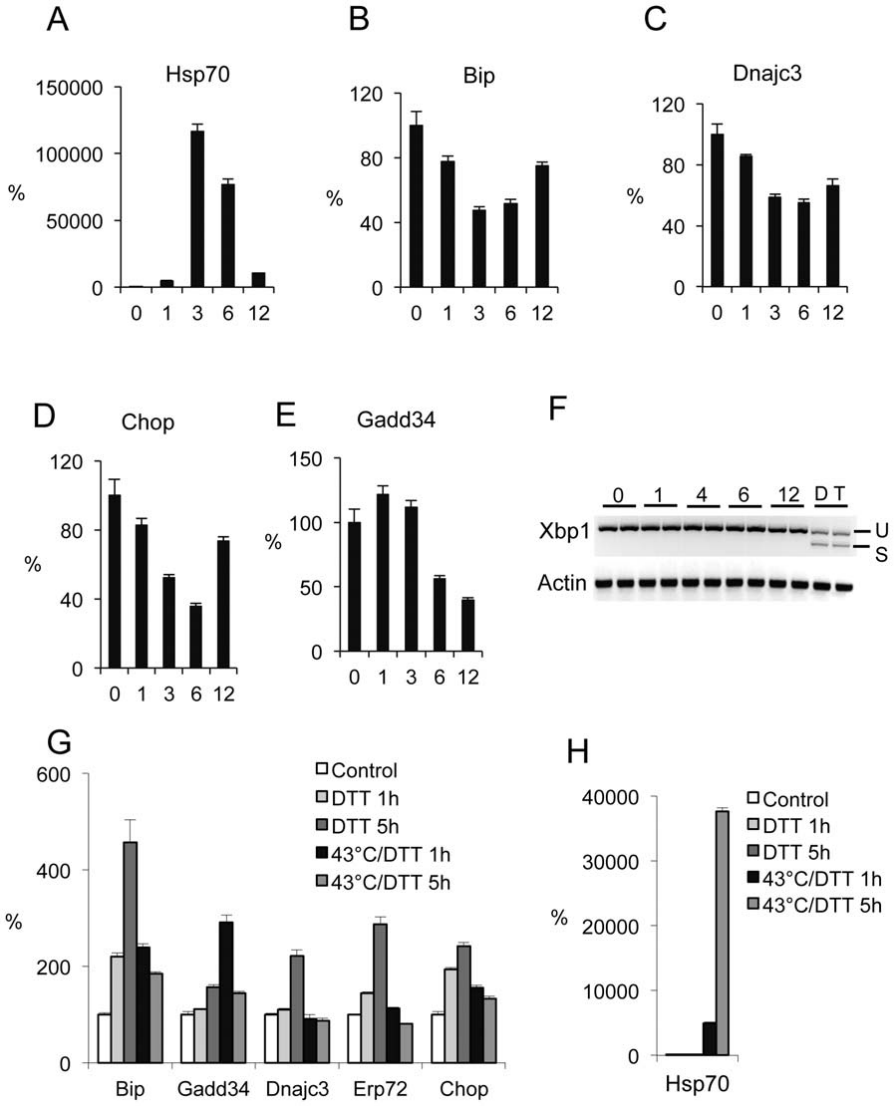
The ER stress response pathway is repressed by a 43°C heat shock in AD293 cells. Relative levels of *Hsp70* (**A**), *Bip* (**B**), *Gadd34* (**C**), *Chop* (**D**), *Dnajc3* (**E**) mRNAs in AD293 cells incubated at 43°C for 1, 3, 6, 12 hours and normalized to mRNAs levels in control cells incubated at 37°C (mean±SEM, n = 3). (**F**) *Xbp1* splicing in AD293 cells at 1, 4, 6, 12 hours after 43°C incubation and control cells incubated at 37°C. Positive controls of *Xbp1* splicing were included as DTT (D) and thapsigargin (T) treatments. *Actin* mRNA levels were used as an internal control. Relative mRNA levels of ER stress related genes (**G**) and *Hsp70* (**H**) in AD293 cells treated with DTT at 37°C or 43°C for 1 or 5 hours and compared to control cells at 37°C (mean±SEM, n = 3).

To confirm earlier studies that the key ER stress genes are not induced by a severe heat shock, AD293 transformed kidney cells were subjected to 43°C heat shock for 1–12 hours. As expected, HSP70 mRNA was potently induced, peaking at 3 hours (120,000 fold), whereas *Bip*, *Chop*, and *Dnajc3* mRNA expression were repressed by approximately 50% at 3 hrs (Fig. 1A–D). *Gadd34* exhibited a very small induction before being repressed at the later time points (Fig. 1E). *Xbp1* splicing, a downstream target of IRE1, was not observed in heat shocked cells in contrast to a robust induction of seen in control cells treated with ER stress inducers DTT or thapsigargin (Fig. 1F). To determine if heat shock would repress the ER stress response, AD293 were treated with the potent ER stress inducer DTT at 43°C and compared to cell treated with DTT at 37°C. DTT treatment elicited the normal induction of *BiP*, *Dnajc3*, *Erp72*, and *Chop* in cells incubated at 37°C but this response to DTT was completely or partially ablated in cells incubated at 43°C (Fig. 1G). *Gadd34*, which is known to be induced by both ER stress and heat shock, was strongly induced in cells treated with DTT at 43°C for one hour. The meteoric induction of *Hsp70* mRNA by heat shock, however, was unfazed by treatment with DTT (Fig. 1H).

**Figure 2:**
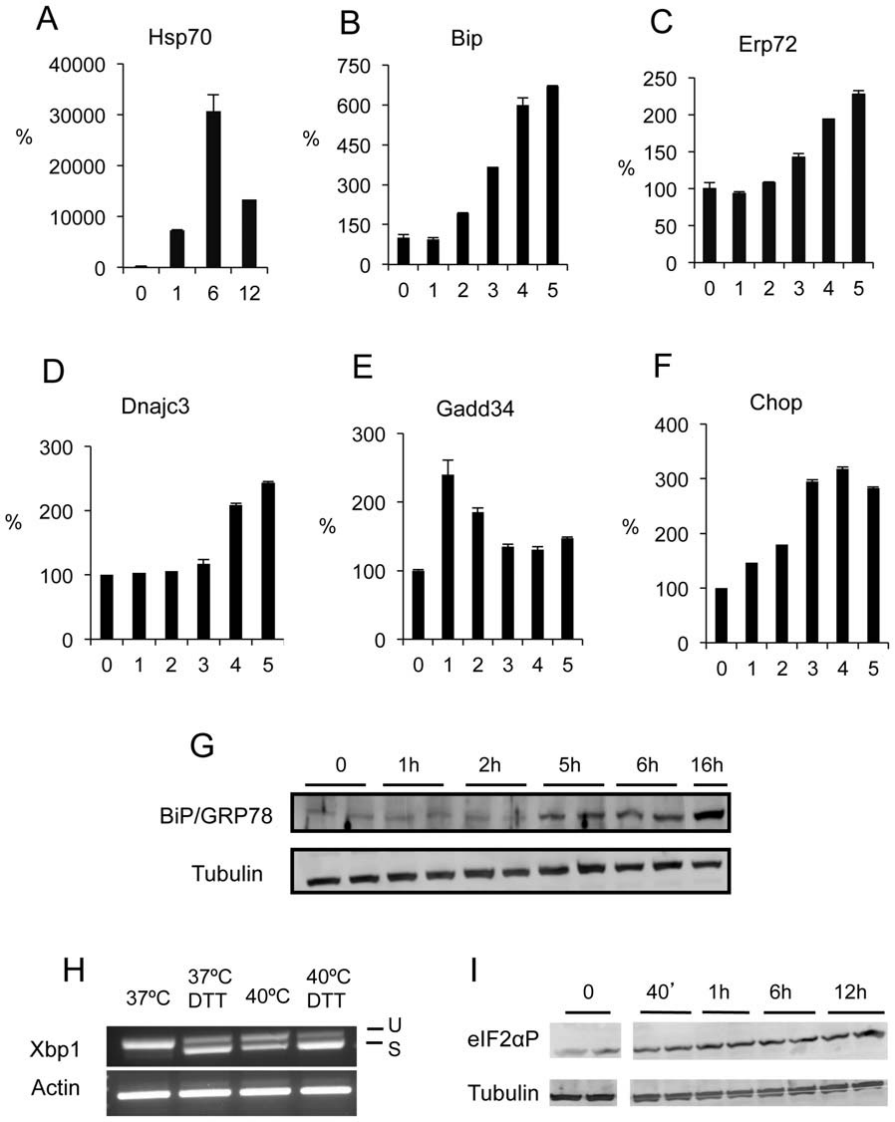
The ER stress response pathway is induced by 40°C hyperthermia in AD293 cells. Relative levels of *Hsp70* (**A**), *Bip* (**B**), *Erp72* (**C**), *Dnajc3* (**D**), *Gadd34* (**E**) and *Chop* (**F**) mRNAs in AD293 cells incubated at 40°C for 1, 2, 3, 4, 5 hours and normalized to expression levels of cells incubated at 37°C (mean±SEM, n = 3). (**G**) BiP/GRP78 protein levels in AD293 cells incubated at 40°C for 0–16 hours with tubulin expression as the loading control. (**H**) *Xbp1* mRNA splicing in AD293 cells incubated at 37°C or 40°C for 6 hours. Positive controls were included as *Xbp1* mRNA splicing induced by DTT treatment. *Actin* mRNA levels were used as an internal control. (**I**) Phosphorylated eIF2α induced by 40°C from 0–12 hours with tubulin protein levels as loading controls.

AD293 cells subjected to 40°C, a temperature corresponding to febrile hyperthermia, also exhibited a strong induction in HSP70 mRNA levels (Fig. 2A). However, in contrast to severe heat shock, *Bip* mRNA was induced 6–7 fold by 5 hours at 40°C (Fig. 2B). BIP protein level (Fig. 2G) closely followed the induction of Bip mRNA. The expression of other ER stress genes was examined including *Erp72*, *Dnajc3*, *Chop*, and *Gadd34* (Fig. 2C–F). *Erp72*, *Dnajc3*, and *Chop* mRNA levels exhibited a similar pattern of delayed induction as seen for *Bip*. However, *Gadd34* exhibited a more rapid response to 40°C hyperthermia, typically peaking at one hour and then declining thereafter. *Xbp1* splicing was also strongly induced although not to the extent seen by treatment with DTT (Fig. 2H). Induction of GADD34, a regulatory subunit of protein phosphatase -1 (PP1), is dependent upon phosphorylation of eIF2α in the ER stress response, and therefore we examined the phosphorylated state of eIF2α during the 40°C heat shock response of AD293 cells. As expected eIF2α phosphorylation was rapidly stimulated by 40 minutes and continued at a high level for 12 hours (Fig. 2I). A similar induction of these markers of the ER stress response was also seen at 39°C (data not shown).

**Figure 3:**
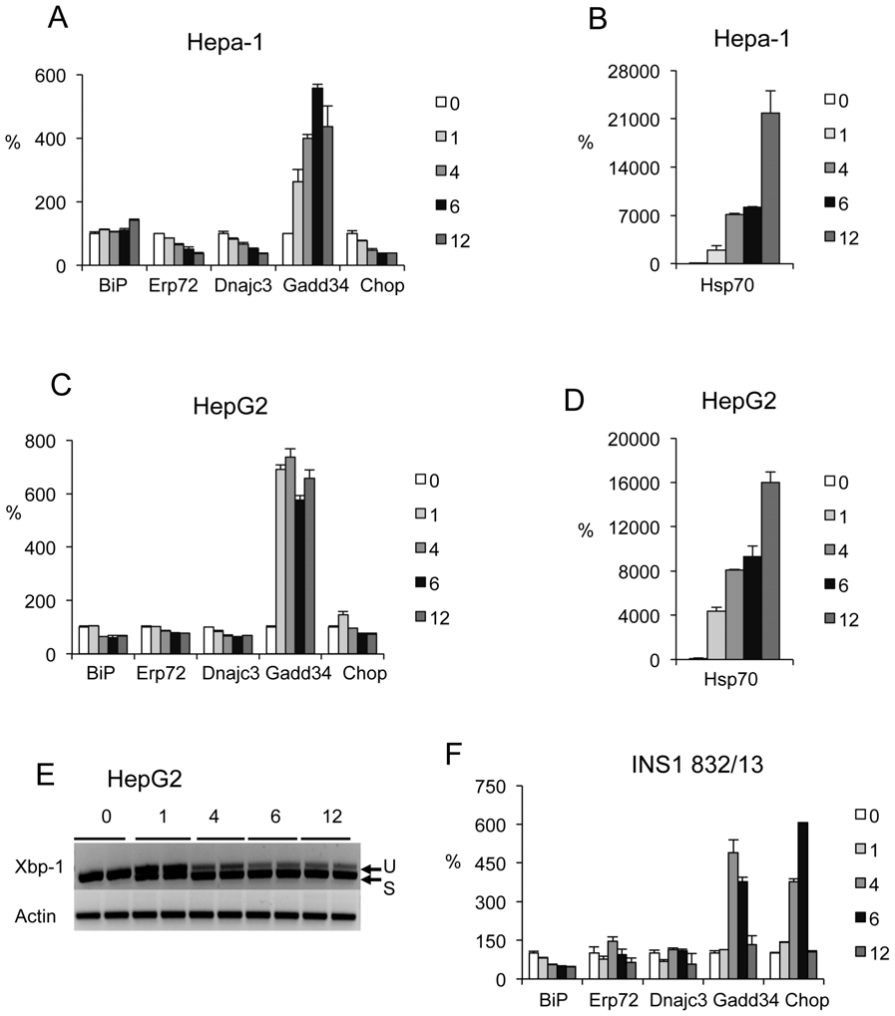
Hepatic and pancreatic beta cell lines display partial induction of ER stress genes in response to hyperthermia. Relative mRNA levels of ER stress related genes (**A** & **C**) and *Hsp70* (**B** & **D**) in Hepa1 and HepG2 cells, respectively, incubated at 40°C for 1, 4, 6, 12 hours and normalized to mRNA levels of cells incubated at 37°C (mean±SEM, n = 3). (**E**) *Xbp1* splicing in HepG2 cells at 0, 1, 4, 6, 12 hours incubated at 40°C. (**F**) Relative mRNA levels of ER stress related genes in INS1 832/13 pancreatic beta cells incubated at 40°C for 1, 4, 6, 12 hours and normalized to mRNA levels of cells incubated at 37°C (mean±SEM, n = 3).

The response of three other cell types to 40°C hyperthermia was determined to assess the generality of these findings. The mRNA expression of the ER stress inducible ER chaperones *Bip*, *Erp72*, and *Dnajc3* were either not induced or substantially declined in human HepG2 and mouse Hepa-1 hepatocyte cell lines (Fig. 3A–D). However, *Gadd34* was strongly induced whereas *Chop* was not. *Xbp-1* RNA splicing was assessed in HepG2 cells and found to be induced to a high level (Fig. 3E) and eIF2α phosphorylation was also induced (data not shown). The pancreatic insulin secreting beta cell line, INS1-832/13 exhibited a strong induction of *Gadd34* and *Chop* but the three ER chaperone genes, *Bip*, *Erp72*, and *Dnajc3* all showed a modest decline in mRNA expression over 12 hours treatment at 40°C (Fig. 3F).

**Figure 4:**
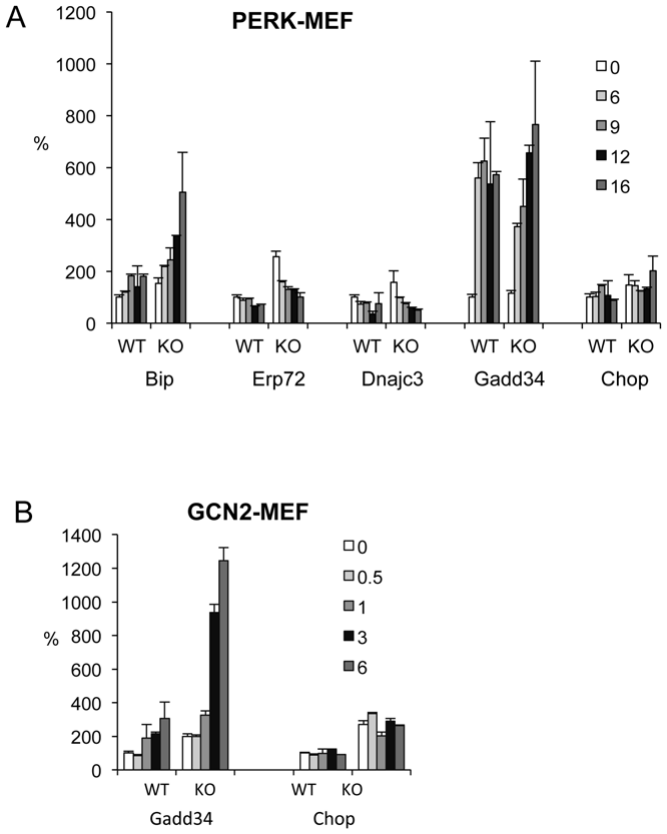
Deficiency of *Perk* or *Gcn2* predisposes mouse embryonic fibroblasts to increased ER stress response induced by hyperthermia. (**A**) Relative mRNA levels of ER stress related genes in *Perk* wildtype (WT) and knockout (KO) MEFs incubated at 40°C for 6, 9, 12, 16 hours and normalized to mRNA levels of cells incubated at 37°C (mean±SEM, n = 3). (**B**) Relative mRNA levels of *Gadd34* and *Chop* in *Gcn2* wildtype (WT) and knockout (KO) MEFs incubated at 40°C for 0.5, 1, 3, 6 hours and normalized to mRNA levels of cells incubated at 37°C (mean±SEM, n = 3).

PERK eIF2α kinase is required for the induction of GADD34 and CHOP in the ER stress response induced pharmacologically by DTT, thapsigargin, or tunicamycin. To determine if induction of *Gadd34* and/or *Chop* by hyperthermia was PERK-dependent, *Perk* KO mouse embryonic fibroblasts (MEFs) were incubated 40°C for 0–16 hours. In wildtype MEF control cells *Bip* mRNA exhibited a modest induction whereas *Erp72* and *Dnajc3*, and *Chop* failed to be induced or were moderately repressed (Fig. 4A). Curiously *Bip* mRNA was even more strongly induced in *Perk* KO MEFs. *Erp72* and Dnajc3 both exhibited higher basal expression and were repressed by hyperthermia. *Gadd34* was strongly induced in both WT and *Perk* KO MEFs, demonstrating that PERK is not required for the induction of *Gadd34*. *Xbp1* RNA splicing was stimulated in both *Perk* KO and WT cells (not shown). GCN2 eIF2α kinase, which is widely expressed in mammalian tissues, was also examined as a candidate for regulating *Gadd34* and *Chop* during hyperthermia. *Gcn2* KO MEFs exhibit higher basal levels of *Gadd34* mRNA and show a 6-fold induction by hyperthermia, whereas *Gcn2* WT MEFs exhibited a more modest 3-fold induction (Fig. 4B). Similarly basal levels of *Chop* are considerably higher in *Gcn2* KO MEFs, but *Chop* is not induced by hyperthermia in either genotype.

## Discussion

Heat shock and ER stress were previously thought to be stimulated under different circumstances, largely because GRP78/BiP was shown at the time of its discovery not to be induced by severe heat shock. We found that a more modest elevation of temperature to 40°C, which corresponds to physiological hyperthermia, elicits an ER stress response in the human AD293 kidney cell line along with a robust induction of *Hsp70* in the absence of a chemical ER stress inducer. The downstream ER stress targets of ATF6 including *Bip*, *Erp72*, and *Dnajc3*, the downstream targets of eIF2α phosphorylation including *Chop* and *Gadd34,* and *Xbp1* mRNA splicing, the downstream target of IRE1 were all induced in AD293 incubated at 40°C. Raising the temperature only 3°C, however, repressed the ER stress response seen at 40°C or in the presence of a chemical inducer of ER stress. These findings are consistent with earlier studies that showed that severe heat shock strongly represses general transcription and translation, and only the heat shock genes are transcribed [23], [24]. Therefore failure to see transcriptional induction of the ER stress genes under severe heat shock, even in the presence of a chemical inducer of ER stress, was expected. However, under more physiologically relevant conditions of febrile hyperthermia, general transcription and translation are not repressed or at least not to the extent seen in at 43°C. Thus under conditions similar to febrile hyperthermia, a heat shock response and ER stress response can occur simultaneously. Induction of both pathways simultaneously may have synergistic effects in reducing cytoplasmic and ER stress as has been suggested in studies in yeast where the heat shock response can relieve ER stress [25].

Although downstream targets of the ATF6 arm of the ER stress pathway were induced in AD293 at 40°C, generally they were not induced in the other wild-type cell types tested including two hepatocyte cell lines, a pancreatic beta cell line, and mouse embryonic fibroblasts. Targets of the PERK and IRE1 arms of the ER stress pathway, however, were generally induced in these other cell types. *Bip* mRNA was modestly induced in *Perk* and *Gcn2* wildtype MEFs and strongly induced in *Perk* KO MEFs. *Chop* mRNA also exhibited considerable variation among cell lines–strongly induced in pancreatic beta cells, repressed in Hepa-1 and unchanged in HepG2 cells. *Gadd34* was consistently induced in all cell lines and typically during the first hour. Although the ER stress pathway has been characterized as an integrated response that requires the activation of all three regulatory arms (IRE1, PERK, and ATF6), there are a number of examples of induction of only one or two of these regulators [26], [27], [28]. Moreover, IRE1 and PERK are known to be activated by physiological stimuli that are unrelated to ER stress or other cellular stresses [8], [11], [29], [30], [31], [32]. We have previously proposed that the normal, non-stress, functions of these regulators are co-opted during ER stress to regulate different pathways to alleviate stress [9].

*Gadd34* expression was shown previously to be induced during ER stress or amino acid deprivation, as mediated by PERK and GCN2, respectively [19], [20], [21]. Curiously, *Gadd34* mRNA was still strongly induced in mouse embryonic fibroblasts deficient for either *Perk* or *Gcn2*. Perhaps one or both of the other two eIF2α kinases, namely PKR and HRI, may be responsible for the induction of *Gadd34* during hyperthermia [33], [34]. Basal or induced levels of some of the ER stress genes were elevated in either *Perk* or *Gcn2* KO MEFs, suggesting that basal expression of PERK and GCN2 provides protection against hyperthermia.

In summary the ER stress response pathway can be fully activated as consequence of hyperthermia in AD293 kidney cells or partially activated in other cell lines. Because the endoplasmic reticulum faces the same challenges of protein folding and quality control during hyperthermia as does the cytoplasm we postulate that the activation of the ER stress pathway in parallel with the heat shock response orchestrate adaptation to febrile hyperthermia that occurs as consequence of disease and infection.

## Materials and Methods

### Cell lines and treatments

AD293 [35] cells and HepG2 [36] cells were cultured in high-glucose DMEM (GIBCO) supplemented with 10% FBS and 1X antibiotic/antimycotic solution (Sigma, Inc.). Hepa1 [37] cells were cultured in high-glucose DMEM with 8% FBS and 1X antibiotic/antimycotic solution. The INS1-832/13 [38] cells were cultured in RPMI-1640 (Mediatech Cellgro) supplemented with 11 mM glucose, 10% FBS, 10 mM HEPES, 1 mM sodium pyruvate, 50 µM β-mercaptoethanol and antibiotic/ antimycotic solution. *Perk*+/+, *Perk*-/-, *Gcn2*+/+ and Gcn2-/- Mouse Embryo Fibroblasts (MEFs) [39], [40] were cultured in high-glucose DMEM (GIBCO) supplemented with 10% FBS, 0.1 mM mercaptoethanol, 10 mM MEM nonessential amino acids (GIBCO) and antibiotic/antimycotic solution. All cell lines were maintained at 37°C in 5% CO_2_ and then switched to a 40°C or 43°C incubator in 5% CO2 during hyperthermia experiments.

### RNA extraction and quantitative RT-PCR

RNA was extracted with Qiagen RNAeasy® Micro Kit (Qiagen) from all cell lines. RNA was quantitated by Quant-It ™ RiboGreen® RNA Assay Kit (Invitrogen). 1 µg RNA was used for reverse transcription with qScript™ cDNA supermix (Quanta) to generate cDNA in a 20 ul reaction volume. Quantitative RT-PCR was performed with qPCR core kit for SYBR® Green I (Eurogentec/AnaSpec) by 7000 Sequence detection system (Applied Biosystems). GAPDH was coamplified with genes of interest as a normalization control. The cycle differences with GAPDH are used to determine the relative intensity of genes of interests. Primers used for real-time PCR are listed in Fig. S1.

### XBP-1 splicing

Human *Xbp1* mRNA from AD293 cells was reverse transcribed to cDNA and amplified by PCR at an annealing temperature at 55°C and cycle number of 40 as previously described [8]. PCR products were run on 2–3% agarose gel to separate spliced *Xbp1* (398 base pairs) and unspliced *Xbp1* (434 base pairs).

### Western blotting

Whole cell lysates were extracted with RIPA buffer containing protease inhibitor cocktail (Sigma), phosphatase inhibitor cocktails 1 and 2 (Sigma) and subjected to Western blot analysis. Primary antibodies for BiP/GRP78 (Santa Cruz), phospho-eIF2α (Cell Signaling) and α tubulin (Sigma) were used.

### Data Analysis

All data are expressed as mean±SEM (n = 3) in arbitrary units normalized to control group as indicated (%). The two-tailed Student's test was used to evaluate statistical differences between the control group and experimental groups.

## Supporting Information

Figure S1 **Primers used for quantitative real-time PCR with ABI 7000 RT PCR System.**
(PDF)
