# Supplementary material for: Hyperthermia Induces the ER Stress Pathway
Source: PLoS One. 2011 Aug 18;6(8):e23740. doi: 10.1371/journal.pone.0023740 (PMC3158104; doi:10.1371/journal.pone.0023740)
Supplement: Figure S1 — Primers used for quantitative real-time PCR with ABI 7000 RT PCR System. (PDF) [file pone.0023740.s001.pdf]

Supplementary Figure S1 Primers used for real-time PCR

Species	Gene	Forward primer	Reverse primer
Human	Hsp70	tttttcgagagtgactcccgtt	cacaggttcgctctgggaag
	Bip	ccttgtcttcagctgtcact	tgcttgatgtatgtcccctta
	Gadd34	tgagcgagaggcaaccagt	tctgcaaattgacttccctgc
	Chop	tgggctacactgagcaccag	cttcgggacacttgccagct
	Dnajc3	ccttcgtactgcagatccacc	gggctcggtattccccttc
	Erp72	aataccaggatgccgctaac	gcaaaggtgtactcagggaa
	GAPDH	ccagggctgcttttaactc	gctcccccctgcaaatga
Mouse	Hsp70	tggtgagccacttcgtgga	tgatgtccttcttgtgcttcctc
	Bip	gcttcgtgtctcctcctgac	taggagtccagcaacaggct
	Gadd34	gtgcagcccaaggtgattct	aggtggccttctatttacccg
	Chop	ccaacagaggtcacacgcac	tgactggaatctggagagcga
	Dnajc3	atgtcagctgcccgtagtgtg	gacctgtgtccatgcagcc
	Erp72	ttccacgtgatggatgttcag	agtcttacgatggcccacca
	GAPDH	ggagcgagaccccactaaca	acatactcagcaccggcctc
Rat	Hsp70	ggtcactgctagctccgtgtt	aacccccacaaatcacaacc
	Bip	acccttactcgggccaaatt	agagcggaacaggtccatgt
	Gadd34	caccacctccccaactttctt	ctacagccccttcacctgca
	Chop	cctgaaagcagaaaccggtc	cctcataccaggcttccagc
	Dnajc3	tcccgaatctgctgatcgtt	ggactatgaagctgcccagg
	Erp72	tctaaccaatcaccgggctg	tcatggtaaggtgccgagg
	GAPDH	caccacaactgcttagccc	tggcatggactgtggtcatg
